# Response to editor’s letter

**DOI:** 10.1186/s43168-023-00198-2

**Published:** 2023-05-10

**Authors:** Ahmad M. Shaddad

**Affiliations:** Chest and Tuberculosis Department, Assiut University, Assiut, Egypt

I am writing this letter in response to the letter to the editor submitted to the *Egyptian Journal of Bronchology* with an inquiry on my recently published paper in your reputable journal under the title “Short-term Evaluation of motor and sensory nerve conduction parameters in COVID-19-associated peripheral neuropathy patients” [1].

I am very glad that our recent publication has attracted the interest of the letter’s author. We declare that we are open-minded to any logical inquiries, and that the debate is the base of scientific work that enriches the knowledge of all sides.

I carefully read the letter to the editor. I concluded that the author has one concern about the methodology of the article, which is that we did not refer to any source of the reference value (RV) of the results of the neurophysiological studies, which is the basis for the diagnosis of the neuropathies associated with our patients’ group, namely the axonal and demyelinating motor and sensory disorders. We used values below the 95 percentile or ± 2 SD of the control group.

Interestingly, the author is aware of the dilemma in interpreting the results of the neurophysiological study. He himself half-answered his inquiry, and I will quote from his own letter, “As these RV are impacted by numerous biological (such as age, gender, body height), physical (such as the temperature of the limb), and technical factors [2, 3], different populations-specific RV of motor and nerve conduction studies have been formulated to be utilized in health centers and research [4, 5]” (lines 32–41).

I will start from where the author has ended. To stratify the dilemma of interpretation of the nerve conduction study, the test is influenced by many variables such as age, gender, height, body mass index [2, 3], and the most important factor, technical and operator variation [4–6].

To be clear, the author’s alleged limitation is statistical rather than scientific, as it will be discussed.

An opening sentence of a major task force of the American Association of Neuromuscular & Electrodiagnostic Medicine (2016) states, “There are not uniform standards for nerve conduction testing across the United States” [7]. The task force established seven criteria to accept studies for reference value for nerve conduction study, including sample size, age, subjects, testing factors, statistical analysis, and presentation of data. They also declare, “Although studies that meet these criteria may be few in number, these criteria can serve as benchmarks for future normative study designs.” They also emphasized in discussing the statistical analysis of normative data of nerve conduction studies that Gaussian statistics cannot be used. Tests for normality and alternative statistical methods that accommodate non-Gaussian data must be used. Methods to correct for non-normality include the following: (1) logarithmic or other appropriate mathematical transformation of the data for analysis or (2) utilization of percentile cutoffs to define thresholds of abnormality” [8, 9].

From the above recommendation, the reference value of the nerve conduction study is a matter of debate in literature worldwide. Using mathematical transformation and percentile cutoffs to interpret abnormalities is a cornerstone of diagnosis.

To our knowledge, from the available data, there is no meta-analysis or large-scale study in Upper Egypt for the reference value of nerve conduction study. I will assume that the author of the letter had done a sufficient search of RV of nerve conduction study in Egypt, and he mentioned a study. Again, I will quote him, “Interestingly, Egypt has developed its own RV for these studies [6]” (lines 42–43).

The referenced study is by Khafagi, Hamdy, Hasan, Ismail, Abdelkader, Saleh, and Mohamed [10], where they study standard norms of nerve conduction study in a normal population of Minia Governorate, Egypt. The study does not meet the above task force criteria for data generalization; surprisingly, the mean age of our study group and their age and gender matched were 45 years in the patient’s group and 43 years in the control group. We enrolled 20 patients and 20 controls. The Khafagi A. T. study only enrolled 19 patients in the age group above 40 years. That is to say, the number of our control group exceeds the enrolled sample size of our age group of patients and control.

The lack of sufficient data about the reference value of nerve conduction study and the debate on the interpretation of results is worldwide in the literature until now. Considerable debate remains about using RVs available in the literature and RVs collected in individual clinical neurophysiology departments [11]. Publicly available RVs for NCS are rare; a recent systematic review only found one set of RVs of sufficient quality for a small number of measurements [12]. In addition, the universal application of publicly available RVs is hampered by the fact that they are likely to differ between clinical neurophysiology departments [13, 14], as they are influenced by factors such as electrode placement and size, filter settings, and temperature. Therefore, *several guidelines recommend that clinics develop their own RVs and methods of diagnosing abnormalities* [7, 15].

According to this perspective, the Neurology Department of Assiut University, Egypt, has adopted a diagnosis of abnormality using a cut-off value of bellow or over 95 percentile and/or ± 2 SD of the control group as a diagnosis of abnormality.

Such a statistical approach is not uncommon. In a study, R. H. Reijntjes and his colleagues [11] developed a mixture module to diagnose abnormality in nerve conduction study and concluded that ± 2 SD of normal is the proper diagnosis of abnormality.

Whether the respectable author of the letter agrees or disagrees with our diagnostic and statistical approach according to the available data in the normal population in Upper Egypt regarding nerve conduction study and to rule out the discrepancy in RV in literature, the variables discussed, and technical and operator falsies, we used our control group results ± 2 SD for diagnosis of abnormality. We must acknowledge that our approach, as presented in the article discussion section, which all results of the control group and patients’ group is concomitant with many Egyptian and non-Egyptian publications in this research field.
